# Maintenance of inequity in the provision of chronic dialysis treatment in Brazil

**DOI:** 10.1590/2175-8239-JBN-2025-0033en

**Published:** 2026-01-09

**Authors:** Fábio Humberto Ribeiro Paes Ferraz, Cibele Isaac Saad Rodrigues, Natan Monsores de Sá

**Affiliations:** 1 Universidade do Distrito Federal, Escola Superior de Ciências da Saúde, Departamento de Graduação em Medicina, Brasília, DF, Brazil.; 2 Pontifícia Universidade Católica, Faculdade de Ciências Médicas e da Saúde, Sorocaba, SP, Brazil.; 3 Universidade de Brasília, Faculdade de Ciências da Saúde, Departamento de Saúde Coletiva, Brasília, DF, Brazil.

**Keywords:** Bioethics, Dialysis, Renal dialysis, Equity, Disparities in health levels, Epidemiology

## Abstract

**Introduction:**

The high rate of people with chronic kidney disease on dialysis is a public health problem, especially in developing countries.

**Objectives:**

To evaluate demographic and socioeconomic changes related to dialysis treatment in Brazil from 2002 to 2019.

**Methods:**

This descriptive, analytical study reviewed retrospective documentary data. A comparative analysis was conducted on demographic, economic, and social trends, as well as changes in dialysis service provision in Brazil between 2002 and 2019. Correlation analysis between Municipal Human Development Index (HDI-M) and the number of dialysis units was performed.

**Results:**

There was an increase in the percentage of the older population (5.3% vs. 9.25%) and in life expectancy at birth (70.8 vs. 75.9 years). The gross domestic product (GDP) increased by 453%; the percentage of investment in public health (below 4%) was stable and the ranking of global Human Development Index decreased (73 vs 84). The increase in the prevalence of patients on chronic maintenance dialysis was greater than the increase in the number of patients in new centers (117.3% vs. 43.9%), with fewer patients receiving treatment in the North and Northeast regions. There was a positive linear correlation between the HDI-M values and the number of dialysis units (R = 0.52; 95% CI: 0.75–0.18; p = 0.006).

**Conclusion:**

Despite Brazil’s strong economic growth and the drastic demographic changes that occurred during the study period, this progress did not translate into a higher investment in health and equitable access to dialysis treatment across the country.

## Introduction

Chronic kidney disease (CKD 5D) is a serious global public health problem due to its increasing prevalence and significant economic impact on health systems^1,2,3^. It is estimated that by 2030, more than 5.4 million individuals worldwide will require some form of renal replacement therapy (RRT), especially in developing countries in Africa and Asia^3^. Access to RRT is marked by global inequality, with only one third of patients – mostly living in high-income countries – having access to these technologies^3^.

Studies have focused on the rapid increase in the incidence of people with CKD on dialysis in developing countries^1,2,3,4^. The progression of kidney diseases is influenced by a multifactorial interplay between traditional factors, including rapid urbanization and increased life expectancy, and nontraditional factors, such as ongoing endemic infectious diseases and poor sanitary conditions^4,5^.

Brazil is a country of continental dimensions, occupying practically half of all of South America, and one of the most populous countries, with more than 212 million people^6^. Despite being among the 15 largest global economies, it has a medium Human Development Index and a high Gini index value, placing it among the 10 most economically unequal countries worldwide^6^.

Brazil is a developing country that plays a leading role in international nephrology due to the large number of patients in maintenance dialysis programs, particularly hemodialysis^4,7^. Such prominence is explained by the magnitude of its public health system – the Unified Health System (SUS) –which is responsible for most of the highly complex medical procedures in the country^8^.

Nearly 10% of the Ministry of Health’s total budget is allocated to supporting patients through the current modalities of RRT (hemodialysis, peritoneal dialysis, and kidney transplantation)^9^. As in many other countries, the main form of RRT in Brazil is intermittent hemodialysis, which is usually performed three times a week for 4 hours in hospital units or satellite centers associated with the SUS^7^.

The annual Brazilian Dialysis Survey was established by the Brazilian Society of Nephrology in 1999, being an important tool for analyzing the situation of maintenance dialysis in Brazil^10,11,12,13^. Data from 2002 showed regional variations in prevalence rates, suggesting inequities in access to dialysis treatment across Brazil^10^.

Despite the Brazilian Dialysis Survey of 2022 and 2023 showing an increase in the prevalence of CKD requiring dialysis (758 vs 771 pmp - patients per million people), the numbers are lower than those in European or even other Latin American countries (such as Chile, Argentina, and Uruguay), which indicates a probable inequity in access to dialysis in Brazil^14^. The estimated total number of patients on maintenance dialysis in Brazil in 2023 was 157,357^11^.

The period between 2002 and 2019 was marked by the publication of the main ordinances and the Resolutions of the Collegiate Board of Directors (RDC), which standardized and regulated dialysis activity throughout the country^15^.

This study analyzed data from 2002 to 2019 to assess whether Brazil’s economic, demographic, and social shifts were reflected in the prevalence of CKD stage 5D and in the equitable provision of dialysis services across the national territory. The period after 2019 was excluded because of the COVID-19 pandemic and its effects on the provision of RRT in Brazil^16^.

## Methods

This descriptive-analytical study used retrospective documentary data from two time periods—2002 and 2019. Publicly accessible data were used to capture demographic, economic, and social changes, while restricted-access data were used to assess dialysis treatment variables. The objective was to analyze transformations that occurred between these two temporal benchmarks.

All variables were selected based on criteria outlined below that referred to these two specific years:

1. Demographic, economic, and social variables:
  - Demographics: estimates of the Brazilian population (total and over 65 years of age) and variation in life expectancy were obtained from the Brazilian Institute of Geography and Statistics (IBGE) website^10,17^.
  - Economic values: values of nominal gross domestic product (GDP) (i.e., corrected for inflation) and percentage of GDP allocated health services (total and public health) were obtained from the World Bank website^18^.
  - Social factors: Brazilian Human Development Index (HDI) and Municipal HDI (HDI-M) were accessed on the United Nations Development Program (UNDP) website^19^.
2. Geographical variables used for dialysis treatment:
  - National data on the number of dialysis centers/clinics, centers/per million people (pmp), and the estimated prevalence of dialysis patients/pmp were obtained from the Brazilian Dialysis Survey for the years 2002 and 2019^10,20^.
  - Municipal data for 2019—including the number and percentage of municipalities offering dialysis services, as well as the number and percentage of dialysis centers in the capitals—were kindly provided by the Brazilian Society of Nephrology (SBN) through its Committee of National and International Registries and Projects.

### Statistical Analysis

Descriptive statistics were used for the absolute and relative frequencies of the quantitative data, which were presented in tables for better visualization.

Quantitative variables were compared between two independent groups according to the nonparametric Mann-Whitney test (MW) and presented as the median and interquartile range.

Linear correlation analysis was conducted using the Spearman correlation coefficient, reported with a 95% confidence interval calculated by the bootstrap method with 1000 replications and interpreted as follows:

- Very strong linear correlation: | coef. | = 0.9–1.0;
- Strong: | coef. | = 0.7–0.9;
- Moderate: | coef. | = 0.4–0.7;
- Weak: | coef. | = 0.2–0.4;
- Very weak: | coef. | = 0.0–0.2

Statistical analyses were performed using R 4.2.2 (R Foundation, Vienna, Austria) and JASP 0.16.4 software. All hypothesis testing were two-tailed, and a p value < 0.05 was considered significant.

### Ethical Aspects

Due to the nature of the study, which was based on retrospective secondary data from publicly available information systems, there was no involvement of human participants. The authors did not access any identifiable individual-level data. Therefore, submission to a Research Ethics Committee and informed consent were not required.

## Results

The main Brazilian demographic, economic and social variables of 2002 and 2019 are presented in Table 1.

**Table 1:** Brazilian demographic, economic and social variables between 2002 and 2019

Parameters	Base Year 2002	Base Year 2019	Variation
1. Demographics
Brazilian population (inhabitants)	174,632,960	210,147,125	20.34%
Elderly population (above 65 years old)	9,712,231	19,525,474	101.04%
Percentage of elderly population/general population	5.30%	9.25%	N.A.
Life expectancy at birth (in years)	70.8	75.9	5.1
2. Economics^*^
GDP (total)	1.32 trillion	7.3 trillion	453%
GDP investment in health^**^ (n – %)	115 billion (8.7%)	711 billion (9.6%)	N.A.
GDP investment in public health (n – %)	46 bi (3.5%)	281 bi (3.8%)	N.A.
3. Social
Global HDI (varies between 0–1)	0.699	0.765	N.A.
World HDI ranking	73°	84°	–11

Abbreviations – GDP: Gross Domestic Product; HDI: Global Human Development Index; N.A.: not applicable.

Notes – *Values in reals;

**including supplementary health expenses.

From a demographic perspective, the older population grew substantially more than the general population between 2002 and 2019 (an increase of 101% versus 20.3%). The proportion of older adults in the total population rose from 5.3% to 9.25%, alongside an increase of 5.1 years in life expectancy at birth—from 70.8 to 75.9 years (Table 1).

Between 2002 and 2019, Brazil’s GDP increased by 453 % (1.32 vs 7.3 trillion reals). However, during this period, the percentage of the GDP allocated to health remained around 8% (115 billion vs 711 billion), while actual public health expenditures accounted for less than 4% of the GDP (46 billion reals – 3.5% vs 281 billion reals – 3.8%). Despite an increase in Brazil’s HDI, the country fell 11 positions in the global HDI ranking (Table 1).

Data regarding HDI-M values, population, and number of dialysis centers nationally and by state between 2002 and 2019 are shown in Table 2.

**Table 2:** Municipal development index (HDI-M) values, population and number of dialysis centers by state between 2002 and 2019

States	Region	2002					2019			
		HDI-M^*^	population	dialysis centers (n)	centers (pmp)	×	HDI-M	population	dialysis centers (n)	centers (pmp)
Acre	N	0.517	586,942	1	1.70	×	0.739	881,935	4	4.54
Amazon	N	0.515	2,961,801	2	0.68	×	0.726	4,144,597	5	1.21
Pará	N	0.518	6,453,683	5	0.77	×	0.704	8,602,865	24	2.79
Roraima	N	0.598	346,871	NA	NA	×	0.749	605,761	2	3.30
Rondônia	N	0.537	1,431,777	2	1.40	×	0.73	1,777,225	7	3.94
Amapá	N	0.577	516,511	1	1.94	×	0.737	845,731	3	3.55
Tocantins	N	0.525	1,207,014	2	1.66	×	0.751	1,572,866	4	2.54
Maranhao	NE	0.476	5,803,224	6	1.03	×	0.694	7,075,181	13	1.84
Piauí	NE	0.484	2,898,223	6	2.07	×	0.706	3,272,447	10	3.06
Ceará	NE	0.541	7,654,535	16	2.09	×	0.744	9,132,858	24	2.63
Rio Grande do Norte	NE	0.552	2,852,784	4	1.40	×	0.742	3,506,853	11	3.14
Paraíba	NE	0.506	3,494,893	7	2.00	×	0.713	4,018,127	11	2.74
Pernambuco	NE	0.544	8,084,667	17	2.10	×	0.74	9,557,517	24	2.51
Alagoas	NE	0.471	2,887,535	8	2.77	×	0.687	3,336,911	14	4.20
Sergipe	NE	0.518	1,846,039	2	1.08	×	0.705	2,298,902	3	1.30
Bahia	NE	0.512	13,323,212	19	1.43	×	0.718	14,872,858	40	2.69
Espírito Santo	SE	0.64	3,201,722	16	5.00	×	0.793	4,018,650	19	4.73
Minas Gerais	SE	0.624	18,343,517	68	3.71	×	0.793	21,168,791	93	4.39
Rio de Janeiro	SE	0.664	14,724,475	69	4.69	×	0.809	17,264,943	83	4.81
São Paulo	SE	0.702	38,177,742	132	3.46	×	0.845	45,919,049	179	3.90
Paraná	S	0.65	9,798,006	42	4.29	×	0.807	11,433,957	49	4.29
Santa Catarina	S	0.674	5,527,707	23	4.16	×	0.826	7,164,788	34	4.75
Rio Grande do Sul	S	0.664	10,408,540	68	6.53	×	0.801	11,377,239	74	6.50
Goiás	CW	0.615	5,210,335	17	3.26	×	0.774	7,020,904	29	4.13
Mato Grosso	CW	0.601	2,604,742	7	2.69	×	0.779	3,484,466	11	3.16
Mato Grosso do Sul	CW	0.613	2,140,624	6	2.80	×	0.777	2,778,986	13	4.68
Distrito Federal	CW	0.725	2,145,839	14	6.52	×	0.859	3,012,718	23	7.63
Brazil		0.692	174,632,960	560	3.21	×	0.765	210,147,125	806	3.84

Abbreviations – N: North; NE: Northeast; SE: Southeast; S: South; CW: Central-West; NA: not available.

Data were grouped by Brazilian macroregion and added to other variables (proportion of centers/pmp, estimated prevalence of dialysis patients/pmp/region and median HDI-M/region), which served as the basis for Table 3.

**Table 3:** Comparison between demographic data, distribution of dialysis centers, prevalence of patients with ckd 5-d in maintenance dialysis and hdi-m values by brazilian macroregion between 2002 and 2019

Macroregions	Base Year 2002	Base Year 2019	Variation (%)
North			
Population (n – %)	13,157,728 (7.7%)	18,430,980 (8.8%)	40.08
dialysis centers (n – %)	13 (2.3%)	47 (6.1%)	261.54
dialysis centers/pmp	0.99	2.55	NA
CKD 5-D prevalence/pmp	109	423	288.07
HDI-M (median)	0.525	0.737	NA
Northeast			
Population (n – %)	48,845,112 (28%)	57,071,654 (27.2%)	16.84
dialysis centers (n – %)	85 (15.2%)	159 (18.6%)	87.06
dialysis centers/pmp	1.74	2.78	NA
CKD 5-D prevalence/pmp	211	594	181.52
HDI-M (median)	0.512	0.713	NA
Southeast			
Population (n – %)	74,447,456 (42.6%)	88,371,433 (42%)	18.70
dialysis centers (n – %)	285 (50.9%)	374 (46.4%)	31.23
dialysis centers/pmp	3.83	4.23	NA
CKD 5-D prevalence/pmp	403	763	89.33
HDI-M (median)	0.65	0.801	NA
South			
Population (n – %)	25,734,253 (14.7%)	29,975,984 (14.2%)	16.48
dialysis centers (n – %)	133 (23.7%)	157 (19.5%)	18.05
dialysis centers/pmp	5.17	5.24	NA
CKD 5-D prevalence/pmp	362	627	73.20
HDI-M (median)	0.664	0.807	NA
Central-West			
Population (n – %)	12,101,540 (7%)	16,297,074 (7.8%)	34.67
dialysis centers (n – %)	44 (7.9%)	76 (9.4%)	72.73
dialysis centers/pmp	3.64	4.66	NA
CKD 5-D prevalence/pmp	285	743	160.70
HDI-M (median)	0.614	0.778	NA
Brazil			
Population (n – %)	174,632,960 (100%)	210,147,125 (100%)	20.34
dialysis centers (n – %)	560 (100%)	806 (100%)	43.93
dialysis centers/pmp	3.21	3.83	NA
CKD 5-D prevalence/pmp	306	665	117.32
Global HDI	0.699	0.765	NA

Abbreviations – HDI-M: Municipal Human Development Index; pmp: per million people; NA: not applicable.

During this 17-year period, the national variation in the prevalence of patients on dialysis/pmp was much greater than the variation in the number of dialysis units (306 to 665 patients – 117.3% vs. 560 to 806 centers – 43.9%, respectively) (Table 3).

The North Region of Brazil showed the highest population growth (13,157,728 vs 18,430,980 – 40.1%), the largest increase in the number of dialysis centers (13 vs 47 – 261.54%), and the greatest rise in the prevalence of CKD 5D patients/pmp (109 vs 423 pmp – 288.1%). It also exhibited the most significant improvement in median HDI-M, from 0.525 to 0.737 (Table 3).

However, the changes in this 17-year period did not geographically alter the percentage of dialysis centers by districts. The number of dialysis centers/pmp was still high in 2019, especially in the Southeast and South regions, (4.23 and 5.24 units/pmp) to the detriment of the North and Northeast (2.55 and 2.78 units/pmp respectively) (Table 3).

The geographical distribution of dialysis centers in Brazil in 2019 is shown in Table 4. The Brazilian states are listed in ascending order of HDI-M values, with a breakdown by state of the following variables: number of dialysis centers, population, number of municipalities, municipalities with dialysis (total and percentage), dialysis centers in capital cities (total and percentage), and proportion of dialysis centers/pmp (Table 4).

**Table 4:** Geographic distribution of dialysis centers throughout the national territory according to increasing hdi-m values (2019)

State	HDI-M 2019	Centers for dialysis	Population (n)	Municipalities (n)	Municipalities with dialysis (n – %)	Dialysis centers Capitals (n – %)	Dialysis centers (pmp)
Alagoas	0.687	*14*	*3,336,911*	*102*	*4 (3.9%)*	*10 (71.4%)*	*4.2*
Maranhão	0.694	*13*	*7,075,181*	*217*	*5 (2.3%)*	*7 (53.8%)*	*1.8*
Pará	0.704	*24*	*8,602,865*	*144*	*11 (7.6%)*	*12 (50%)*	*2.8*
Sergipe	0.705	*3*	*2,298,902*	*75*	*1 (1.3%)*	*3 (100%)*	*1.3*
Piauí	0.706	*10*	*3,272,447*	*224*	*5 (2.2%)*	*6 (60%)*	*3.0*
Paraíba	0.713	*11*	*4,018,127*	*223*	*3 (1.3%)*	*6 (54.5%)*	*2.7*
Bahia	0.718	*40*	*14,872,858*	*417*	*24 (5.7%)*	*13 (32.5%)*	*2.7*
Amazonas	0.726	*5*	*4,144,597*	*62*	*1 (1.6%)*	*5 (100%)*	*1.2*
Rondônia	0.73	*7*	*1,777,225*	*52*	*5 (9.6%)*	*3 (42.8%)*	*3.9*
Amapá	0.737	*3*	*845,731*	*16*	*1 (6.2%)*	*3 (100%)*	*3.5*
Acre	0.739	*4*	*881,935*	*22*	*1 (4.5%)*	*4 (100%)*	*4.5*
Pernambuco	0.74	*24*	*9,557,517*	*185*	*11 (5.9%)*	*14 (58.3%)*	*2.5*
Rio Grande do Norte	0.742	*11*	*3,506,853*	*167*	*7 (4.2%)*	*4 (36.4%)*	*3.1*
Ceará	0.744	*24*	*9,132,858*	*184*	*13 (7.1%)*	*11 (45.8%)*	*2.6*
Roraima	0.749	*2*	*605,761*	*15*	*1 (6.7%)*	*2 (100%)*	*3.3*
Tocantins	0.751	*4*	*1,572,866*	*139*	*3 (2.2%)*	*2 (50%)*	*2.5*
Goiás	0.774	29	7,020,904	246	15 (6.1%)	12 (41.4%)	*4.1*
Mato Grosso do Sul	0.777	13	2,778,986	79	7 (8.9%)	6 (46.1%)	*4.7*
Mato Grosso	0.779	*11*	*3,484,466*	*141*	*8 (5.7%)*	*4 (36.4%)*	*3.1*
Espírito Santo	0.793	19	4,018,650	78	9 (11.5%)	4 (21.1%)	*4.7*
Minas Gerais	0.793	93	21,168,791	853	58 (6.8%)	15 (16.15)	*4.4*
Rio Grande do Sul	0.801	74	11,377,239	497	46 (9.2%)	15 (20.3%)	*6.5*
Paraná	0.807	49	11,433,957	399	28 (7%)	13 (26,5%)	*4.3*
Rio de Janeiro	0.809	83	17,264,943	92	30 (32.6%)	41 (49.4%)	*4.8*
Santa Catarina	0.826	34	7,164,788	295	23 (7.8%)	4 (11.8%)	*4.7*
São Paulo	0.845	179	45,919,049	645	84 (13%)	52 (29%)	*3.9*
Distrito Federal^*^	0.859	23	3,012,718	1	1 (100%)	18 (78.2%)	*7.6*
Brazil	0.765	806	210,147,125	5570	405 (7.3%)	289 (50.2%)	*3.8*

Sixteen (16) of Brazil’s 27 states had state-level HDI-M values below the national average of 0.765, including all states in the North and Northeast regions. In five states—Sergipe, Amazonas, Amapá, Acre, and Roraima—only the state capital, had a dialysis center (Table 4).

Regarding the national data, only 7.3% of Brazilian municipalities (405 out of 5570) had dialysis units, at a proportion of 3.8 centers/pmp. Furthermore, more than half of these centers (289 units, 50.2%) were located in state capitals (Table 4).

Table 5 shows the comparison between states with HDI-M lower (North and Northeast) and higher (Midwest, Southeast and South) than the national HDI with respect to the following variables: number of dialysis centers, population, total number of municipalities, municipalities with dialysis, concentration of centers in state capitals and proportion of dialysis centers/pmp.

**Table 5:** Comparison between the geographical distribution and number of dialysis centers in states with hdi-m values above and below the national average

Parameters	Lower HDI-M (n = 16)	Higher HDI-M (n = 11)	p value^*^
Dialysis centers (n – %)	199	607	0.001
Population (n – %)	75,502,634	134,644,491	0.036
Municipalities (n – %)	2244	3326	0.132
Cities with dialysis (n – %)	96 (23.7%)	309 (76.3%)	0.003
Number of dialysis centers in capital cities (n – %)	105 (52.7%)	184 (30.3%)	0.001
Number of dialysis centers/pmp	2.8	4.7	<0.001

Note – *P value by the Mann-Whitney test.

Regions with lower HDI-M (North and Northeast) had significantly fewer dialysis centers, fewer municipalities with dialysis, fewer dialysis centers/pmp, and a greater concentration of centers in the capitals (Table 5).

**Figure 1:**
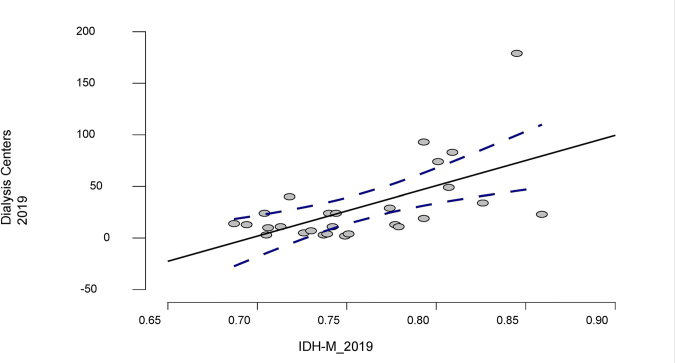
A moderately significant and positive correlation was found between the Human Development Index (HDI-M) 2019 score and the number of dialysis centers in the same year (Spearman correlation coefficient = 0.52; 95% CI: 0.75–0.18; p = 0.006).

A positive linear correlation was observed between the HDI-M and the number of dialysis centers (Spearman correlation coefficient = 0.52; 95% CI: 0.75–0.18; p = 0.006) as shown in Figure 1.

## Discussion

The demographic variations presented in Table 1 reflect the accelerated aging of the Brazilian population. This finding aligns with Global Burden of Disease studies conducted in Brazil, which have highlighted the rise of chronic noncommunicable diseases (CNCDs)—including kidney disease requiring dialysis—as major contributors to morbidity and mortality^21,22,23^. It is estimated that two-thirds of patients on RRT in Brazil have underlying conditions such as arterial hypertension and diabetes mellitus^11,12^.

These findings led to the elaboration of the Strategic Action Plan to Combat Diseases in Brazil 2011-2022^24^ and, more recently, the Strategic Action Plan for Coping with Chronic Diseases and Noncommunicable Diseases (CNCDs) in Brazil 20212030^25^, both of which reinforce the need to create lines of care for the surveillance, information, monitoring, and promotion of comprehensive care for CNCDs. However, important inequities persist both in access to basic health units and in the early diagnosis of CNCDs, especially in the most vulnerable regions of the country^26,27^. An estimated 10% of the world’s population has some degree of loss of renal function; however, a recent survey showed that only 2% of the Brazilian population recognizes this condition, clearly indicating underdiagnosis^28^.

In this context, further changes are still needed within the Brazilian SUS to address CNCDs, which often follow a prolonged, oligosymptomatic course. The system must continue shifting away from the hospital-centered and curative model—originally shaped during the era of infectious disease control—toward a new model centered on health promotion and disease prevention, grounded in continuous, transdisciplinary, and team-based care^29^.

The drastic demographic changes related to aging were not accompanied by a higher percentage of direct investment in public health. The growth of the aging population in contemporary societies creates enormous challenges that need to be addressed during the formulation and planning of state-level public policies; furthermore, promotion, prevention, and care should be directed to the specific needs of this population, building a network that can offer social protection services and actions^30^.

In addition to limited investments in health, the 17-year period did not show improvements in the equitable distribution of income and education (measured by the global HDI), despite economic growth (reflected in the increase in the GDP). Among the countries with universal public health care systems, Brazil has one of the lowest percentages of public expenditures of the GDP, which has persisted over time and provides reasons for the utilitarian mantra that universal health care systems represent, in fact, an “obstacle” to economic growth^31^.

The HDI is a synthetic measure of long-term progress in three basic dimensions of human development. It was created to expand the reductionist economic view of development provided by the per capita GDP. Thus, although still subject to criticism, it does include education and health in addition to income. This index, therefore, is a counterpoint to GDP and is widely used as an indicator^19^.

Tables 2, 3, 4, and 5 and Figure 1 were built and designed based on state HDI-M values. Given Brazil’s continental size and significant regional disparities, the United Nations Development Program in Brazil (UNDP-Brazil), in partnership with the Institute for Applied Economic Research (IPEA) and the João Pinheiro Foundation, developed the HDI-M to assess human development at the municipal level across the country^19^. The calculation of the HDI-M is a methodological adjustment of the global HDI, following its same three dimensions—longevity, education, and income—based on national data^19^.

Table 3 shows that between 2002 and 2019, the number of dialysis centers increased from 560 to 806 units, corresponding to a 43.9% rise. Over the same period, the prevalence of CKD stage 5D/pmp increased from 306 to 665, an increase of 117.3%. Although both indicators increased, the expansion in dialysis centers was proportionally lower than the increase in patient prevalence.

Furthermore, the growth in the number of dialysis centers in this 17-year period was insufficient to reverse the indisputable regional asymmetries (Table 3), with a predominance of dialysis and kidney transplant units in the Southeast and South regions to the detriment of the North, Northeast, and Central-West regions. This asymmetry is not purely demographic; athough the Northeast region is the second most populous region in the country, it has few centers than the South region, thus demonstrating the economic nature of the distribution of centers.

The period from 2002 to 2019 was marked by significant economic growth but also by financial crises and political instability, especially between 2014 and 2016. Regarding health policies, there was a progressive expansion of primary care (through programs such as the Family Health Strategy and the *Mais Médicos* Program) and advances in health surveillance and control of endemic diseases. The expansion of public services occurred concomitantly with the strengthening of the private sector, limiting the consolidation of a truly Universal Health System and perpetuating underfunding and inequalities^32^.

One of the explanations for the small growth in dialysis centers is undoubtedly the chronic underfunding of the system. Between 2002 and 2017, the amount paid by the SUS per hemodialysis session increased by R$91.26 (R$102.94 to R$194.20 per session), which corresponds to an average variation of approximately 5.8%/year, below most of the inflation indices commonly used in Brazil, such as the General Consumer Price Index (IPCA) and General Market Price Index (IGP-M)^15^.

The data in Tables 4 and 5 show severe asymmetry in the geographical distribution of dialysis centers throughout the country, according to increasing HDI-M values for the year 2019. Five states (Roraima, Amapá, Acre, Amazonas and Sergipe) offer dialysis care only in their capitals; moreover, in Roraima, there are only two treatment centers.

Table 5 shows the comparison between states with HDI-M values higher and lower than the national HDI and the obvious inequity. The states with lower HDI-M values (mostly states in the North and Northeast) have statistically fewer dialysis centers, fewer municipalities with dialysis services, fewer dialysis centers pmp, and a greater concentration of centers in state capitals, corroborating that the geographical disparity was maintained in the 17-year study period. Figure 1 shows a positive and significant linear correlation between increasing HDI-M values (higher in the South and Southeast regions) and a greater offer of dialysis centers.

The first anual Brazilian Dialysis Survey was established by the Brazilian Society of Nephrology in 1999^13^. However, the first study that showed the inequities in the offer of dialysis treatment in the country and its direct correlation with state GDP was that of Romāo-Júnior, who analyzed the 2002 Brazilian Dialysis Survey^10^. Subsequent articles studied specific issues related to interstate geographical disparities in access to dialysis^33^. There are inequities in the offer of kidney transplantation^34^, spatial disparities in the offer of health care equipment (such as dialysis machines, magnetic resonance imaging equipment and bone densitometers^35^, and even differences in survival between patients undergoing dialysis through the SUS and those undergoing dialysis through private health insurance plans^36^. The use of the HDI-M tool is not new in Brazilian studies, which show the correlation of the index with the prevalence of infectious diseases^37,38,39^ or even CNCD^40^.

This is one of the first Brazilian studies to thoroughly analyze inequities in the offer of dialysis, revealing demographic, economic, and social changes relative to SUS underfunding over a significant period of time (17 years). The HDI-M values were used as an analysis tool to demonstrate the temporal maintenance of inequity in the offer of dialysis in the country.

One of the limitations of this study is that data from the Brazilian Dialysis Survey were used as an analysis tool^20^. It is important to highlight that the data are based on the centers registered with the Brazilian Society of Nephrology (SBN), which is not a mandatory reporting system. Therefore, the actual number of centers may differ, with the possibility of underestimation (due to lack of registration) or overestimation (if centers have closed but were not removed from the database).

In fact, as data are based on a voluntary survey completed by dialysis centers, the calculated prevalence is approximately equal to the number of patients on dialysis, not individualized, and often subject to underreporting because it is non-mandatory. This situation has encouraged some researchers to use other data sources, such as the issuance of APAC/TRS (Authorization of Complexity Discharge Procedures/Replacement Renal Therapy)^41^.

A recent article using data from the Brazilian Public Health System’s 2023 database complements the data from our study, as it points out – in a post-COVID scenario – that patients from the North, Northeast, and Central-West regions travel greater distances for hemodialysis treatment than patients from the South and Southeast regions^42^.

## Conclusion

There were important changes in the Brazilian sociodemographic aspects in the analyzed period (2002 to 2019), especially regarding the growth of the older population. However, these changes did not translate into an increase in the percentage of GDP allocated to health care or, even less, in the nationwide equitable offer of dialysis, even with the clear increase in the prevalence of CKD 5-D. In addition, regional inequalities are tacit, with dialysis centers concentrated in regions with higher income and in states with higher HDI-M values.

## Data Availability

The entire dataset supporting the findings of this study is available upon request to the corresponding author.
