# Supplementary material for: Maintenance of inequity in the provision of chronic dialysis treatment in Brazil
Source: J Bras Nefrol. 2026 Jan 9;48(1):e20250033. doi: 10.1590/2175-8239-JBN-2025-0033en (PMC12788823; doi:10.1590/2175-8239-JBN-2025-0033en)
Supplement: Translation

# Translation: Manutenção da iniquidade na oferta do tratamento de diálise crônica no Brasil

**DOI:** 10.1590/2175-8239-JBN-2025-0033pt

**Authors:** Fábio Humberto Ribeiro Paes Ferraz, Cibele Isaac Saad Rodrigues, Natan Monsores de Sá

**Affiliations:** 1 Universidade do Distrito Federal, Escola Superior de Ciências da Saúde, Departamento de Graduação em Medicina, Brasília, DF, Brasil.; 2 Pontifícia Universidade Católica, Faculdade de Ciências Médicas e da Saúde, Sorocaba, SP, Brasil.; 3 Universidade de Brasília, Faculdade de Ciências da Saúde, Departamento de Saúde Coletiva, Brasília, DF, Brasil.

**Keywords:** Bioética, Diálise, Diálise renal, Equidade, Disparidades nos níveis de saúde, Epidemiologia

## Abstract

**Introdução:**

A elevada taxa de indivíduos com doença renal crônica em diálise constitui um problema de saúde pública, especialmente nos países em desenvolvimento.

**Objetivos:**

Avaliar as mudanças demográficas e socioeconômicas relacionadas ao tratamento dialítico no Brasil entre 2002 e 2019.

**Métodos:**

Estudo descritivo e analítico com revisão de dados documentais retrospectivos. Foi realizada uma análise comparativa das tendências demográficas, econômicas e sociais, bem como das alterações na oferta de serviços de diálise no Brasil entre 2002 e 2019. Realizouse, ainda, uma análise de correlação entre o Índice de Desenvolvimento Humano Municipal (IDH-M) e o número de unidades de diálise.

**Resultados:**

Houve um aumento no percentual da população idosa (5,3% vs. 9,25%) e na expectativa de vida ao nascer (70,8 vs. 75,9 anos). O produto interno bruto (PIB) aumentou 453%; o percentual de investimento em saúde pública permaneceu estável (abaixo de 4%) e o ranking do Indice de Desenvolvimento Humano Global apresentou redução (73 vs 84). O aumento na prevalência de pacientes em diálise crônica de manutenção foi superior ao aumento no número de pacientes em novos centros (117,3% vs. 43,9%), com menos pacientes recebendo tratamento nas regiões Norte e Nordeste. Houve correlação linear positiva entre os valores do IDH-M e o número de unidades de diálise (R = 0,52; IC 95%: 0,75–0,18; p = 0,006).

**Conclusão:**

O forte crescimento econômico do Brasil e as drásticas mudanças demográficas ocorridas durante o período do estudo nāo se traduziram em maiores investimentos na saúde e acesso equitativo ao tratamento dialítico em todo o país.

## Introdução

A doença renal crônica dialítica (DRC estágio 5D) constitui um problema global de saúde pública devido à sua crescente prevalência e ao significativo impacto econômico sobre os sistemas de saúde^1,2,3^. Estima-se que, até 2030, mais de 5,4 milhões de indivíduos em todo o mundo irão demandar alguma forma de terapia renal substitutiva (TRS), especialmente nos países em desenvolvimento (sobretudo na África e na Ásia)^3^. O acesso a TRS é marcado pela desigualdade global , com apenas um terço dos pacientes - em sua maioria residentes em países de alta renda, tendo acesso a tais tecnologias^3^.

Estudos têm se concentrado no rápido aumento da incidência de indivíduos com DRC em diálise nos países em desenvolvimento^1,2,3,4^. A progressão das doenças renais é influenciada por uma interação multifatorial entre fatores tradicionais (incluindo a rápida urbanização e o aumento da expectativa de vida), e fatores não tradicionais (como doenças infecciosas endêmicas e condições sanitárias precárias)^4,5^.

O Brasil é um país de dimensões continentais, ocupando praticamente metade de toda a América do Sul, e figurando entre os países mais populosos do mundo, com uma população de mais de 212 milhões de habitantes^6^. Apesar de estar entre as 15 maiores economias globais, apresenta um valor de Índice de Desenvolvimento Humano Global (IDH) médio e um valor elevado no índice de *Gini,* o que o coloca entre os 10 países com maior desigualdade econômica no mundo^6^.

O Brasil é um país em desenvolvimento que desempenha papel de destaque na nefrologia internacional devido ao grande número de pacientes em programas de diálise de manutenção, particularmente em hemodiálise^4,7^. Tal protagonismo se explica pela magnitude de seu sistema público de saúde – o Sistema Único de Saúde (SUS) –, responsável pelo financiamento da maior parte dos procedimentos médicos de alta complexidade no país^8^.

Cerca de 10% do orçamento total do Ministério da Saúde é destinado ao suporte de pacientes por meio das modalidades atuais de TRS disponíveis (hemodiálise, diálise peritoneal e transplante renal)^9^. Assim como em muitos outros países, a principal forma de TRS no Brasil é a hemodiálise intermitente, geralmente realizada três vezes por semana, comduração de quatro horas, em unidades hospitalares ou centros satélites vinculados ao SUS^7^.

O Censo Brasileiro de Diálise (CBD) foi instituído pela Sociedade Brasileira de Nefrologia em 1999, constituindo-se em importante ferramenta para a análise da situação da diálise de manutenção no Brasil^10,11,12,13^. Dados de 2002 já revelavam variações regionais nas taxas de prevalência, sugerindo desigualdades no acesso ao tratamento dialítico em todo o território brasileiro^10^.

Embora os Censos Brasileiros de Diálise de 2022 e 2023 tenham demonstrado um aumento na prevalência de DRC estágio 5D (758 vs. 771 pmp - pacientes por milhão de pessoas), os números são inferiores aos observados na Europa ou mesmo em outros países da América Latina (como Chile, Argentina e Uruguai), o que indica uma provável desigualdade no acesso à diálise no Brasil^14^. O número total estimado de pacientes em diálise de manutenção no Brasil em 2023 foi de 157.357^11^.

O período entre 2002 e 2019 foi marcado pela publicação das principais portarias e das Resoluções de Diretoria Colegiada (RDC), que padronizaram e regulamentaram a atividade dialítica em todo o país^15^.

Este estudo teve como objetivo analisar os dados de 2002 a 2019 visando avaliar se as mudanças econômicas, demográficas e sociais do Brasil se refletiram no aumento da prevalência de DRC estágio 5D e na prestação equitativa de serviços de diálise em todo o território nacional. O período após 2019 foi excluído em razão da pandemia de COVID-19 e seu impacto negativo na prestação de TRS no Brasil^16^.

## Métodos

Este estudo descritivo-analítico utilizou dados documentais retrospectivos de dois períodos: 2002 e 2019. Dados de acesso público foram utilizados para capturar mudanças demográficas, econômicas e sociais, enquanto dados de acesso restrito foram utilizados para avaliar variáveis relacionadas ao tratamento de diálise. O objetivo foi analisar as transformações ocorridas entre esses dois marcos temporais.

Todas as variáveis foram selecionadas com base nos critérios descritos a seguir, referentes a esses dois anos específicos:

1. Variáveis demográficas, econômicas e sociais:
  - Dados demográficos: as estimativas da população brasileira (total e com mais de 65 anos) e a variação na expectativa de vida foram obtidas no site do Instituto Brasileiro de Geografia e Estatística (IBGE)^10,17^.
  - Valores econômicos: os valores do produto interno bruto (PIB) nominal (ou seja, corrigido pela inflação) e o percentual do PIB destinado aos serviços de saúde (total e saúde pública) foram obtidos no site do Banco Mundial^18^.
  - Fatores sociais: o Índice de Desenvolvimento Humano (IDH) brasileiro e o IDH municipal (IDH-M) foram consultados no site do Programa das Nações Unidas para o Desenvolvimento (PNUD)^19^.
2. Variáveis geográficas utilizadas para o tratamento dialítico:
  - Dados nacionais sobre o número de centros/clínicas de diálise, centros/por milhão de pessoas (pmp) e a prevalência estimada de pacientes em diálise/pmp foram obtidos a partir do Censo Brasileiro de Diálise para os anos de 2002 e 2019^10,20^.
  - Dados municipais de 2019 — incluindo o número e o percentual de municípios que oferecem serviços de diálise, bem como o número e o percentual de centros de diálise nas capitais — foram gentilmente fornecidos pela Sociedade Brasileira de Nefrologia (SBN) por meio de seu Comitê de Registros e Projetos Nacionais e Internacionais.

### Análise Estatística

Foram utilizadas estatísticas descritivas para as frequências absolutas e relativas dos dados quantitativos, que foram apresentados em tabelas para melhor visualização.

As variáveis quantitativas foram comparadas entre dois grupos independentes de acordo com o teste não paramétrico de *Mann-Whitney* (MW) e apresentadas como mediana e intervalo interquartil.

A análise de correlação linear foi realizada por meio do coeficiente de correlação de *Spearman*, relatado com intervalo de confiança de 95%, calculado pelo método *bootstrap* com 1000 réplicas e interpretado da seguinte forma:

- Correlação linear muito forte: | coeficiente | = 0,9–1,0;
- Forte: | coeficiente | = 0,7–0,9;
- Moderado: | coeficiente | = 0,4–0,7;
- Fraco: | coeficiente | = 0,2–0,4;
- Muito fraco: | coeficiente | = 0,0–0,2

As análises estatísticas foram realizadas utilizando os softwares R 4.2.2 (*R Foundation*, Viena, Áustria) e JASP 0.16.4. Todos os testes de hipóteses foram bicaudais, e um valor de p < 0,05 foi considerado significativo.

### Aspectos Éticos

Devido à natureza do estudo, baseado em dados secundários retrospectivos provenientes de sistemas de informação disponíveis publicamente, não houve envolvimento de participantes humanos. Os autores não tiveram acesso a quaisquer dados individuais identificáveis. Portanto, não foi necessário submeter o estudo a um Comitê de Ética em Pesquisa e nem necessidade de aplicação de termo de consentimento livre e esclarecido.

## Resultados

As principais variáveis demográficas, econômicas e sociais do Brasil para os anos de 2002 e 2019 estão apresentadas na Tabela 1.

**Tabela 1:** Variáveis demográficas, econômicas e sociais do brasil entre 2002 e 2019

Parâmetros	Ano Base 2002	Ano Base 2019	Variação
1. Demográficos
População brasileira (habitantes)	174.632.960	210.147.125	20,34%
População idosa (acima de 65 anos)	9.712.231	19.525.474	101,04%
Percentual da população idosa/população geral	5,30%	9,25%	N.A.
Expectativa de vida ao nascer (em anos)	70,8	75,9	5,1
2. Econômicos^*^
PIB (total)	1,32 trilhão	7,3 trilhões	453%
Investimento do PIB em saúde** (n – %)	115 bilhões (8,7%)	711 bilhões (9,6%)	N.A.
Investimento do PIB em saúde pública (n – %)	46 bi (3,5%)	281 bi (3,8%)	N.A.
3. Sociais
IDH global (varia entre 0 e 1)	0,699	0,765	N.A.
Ranking mundial do IDH	73°	84°	-11

Abreviaturas – PIB: Produto Interno Bruto; IDH: Índice de Desenvolvimento Humano; N.A.: Não Aplicável.

Notas – *Valores em reais;

**incluindo despesas suplementares com saúde.

De uma perspectiva demográfica, a população idosa apresentou um crescimento exponencialmente maior do que a população geral entre 2002 e 2019 (aumento de 101% vs. 20,3%). A percentagem de idosos na população total aumentou de 5,3% para 9,25%, juntamente com um incremento de 5,1 anos na expectativa de vida ao nascer — de 70,8 para 75,9 anos (Tabela 1).

Entre 2002 e 2019, o PIB do Brasil aumentou 453% (1,32 vs. 7,3 trilhões de reais). No entanto, durante esse período, o percentual do PIB destinado à saúde manteve-se em torno de 8% (115 bilhões vs. 711 bilhões), enquanto os gastos reais com saúde pública representaram menos de 4% do PIB (46 bilhões de reais – 3,5% vs. 281 bilhões de reais – 3,8%). Apesar de um aumento no IDH global do Brasil, o país caiu 11 posições no ranking mundial comparativamente (Tabela 1).

Os dados relativos aos valores do IDH-M, população e número de centros de diálise em nível nacional e por estado, entre 2002 e 2019, são apresentados na Tabela 2.

**Tabela 2:** Valores do índice de desenvolvimento humano municipal (idh-m), população e número de centros de diálise por estado entre 2002 e 2019

Estados	Região	2002					2019			
		IDH-M^*^	população	centros de diálise (n)	centros (pmp)	×	IDH-M	população	centros de diálise (n)	centros (pmp)
Acre	N	0,517	586.942	1	1,70	×	0,739	881.935	4	4,54
Amazonas	N	0,515	2.961.801	2	0,68	×	0,726	4.144.597	5	1,21
Pará	N	0,518	6.453.683	5	0,77	×	0,704	8.602.865	24	2,79
Roraima	N	0,598	346.871	NA	NA	×	0,749	605.761	2	3,30
Rondônia	N	0,537	1.431.777	2	1,40	×	0,73	1.777.225	7	3,94
Amapá	N	0,577	516.511	1	1,94	×	0,737	845.731	3	3,55
Tocantins	N	0,525	1.207.014	2	1,66	×	0,751	1.572.866	4	2,54
Maranhão	NE	0,476	5.803.224	6	1,03	×	0,694	7.075.181	13	1,84
Piauí	NE	0,484	2.898.223	6	2,07	×	0,706	3.272.447	10	3,06
Ceará	NE	0,541	7.654.535	16	2,09	×	0,744	9.132.858	24	2,63
Rio Grande do Norte	NE	0,552	2.852.784	4	1,40	×	0,742	3.506.853	11	3,14
Paraíba	NE	0,506	3.494.893	7	2,00	×	0,713	4.018.127	11	2,74
Pernambuco	NE	0,544	8.084.667	17	2,10	×	0,74	9.557.517	24	2,51
Alagoas	NE	0,471	2.887.535	8	2,77	×	0,687	3.336.911	14	4,20
Sergipe	NE	0,518	1.846.039	2	1,08	×	0,705	2.298.902	3	1,30
Bahia	NE	0,512	13.323.212	19	1,43	×	0,718	14.872.858	40	2,69
Espírito Santo	SE	0,64	3.201.722	16	5,00	×	0,793	4.018.650	19	4,73
Minas Gerais	SE	0,624	18.343.517	68	3,71	×	0,793	21.168.791	93	4,39
Rio de Janeiro	SE	0,664	14.724.475	69	4,69	×	0,809	17.264.943	83	4,81
São Paulo	SE	0,702	38.177.742	132	3,46	×	0,845	45.919.049	179	3,90
Paraná	S	0,65	9.798.006	42	4,29	×	0,807	11.433.957	49	4,29
Santa Catarina	S	0,674	5.527.707	23	4,16	×	0,826	7.164.788	34	4,75
Rio Grande do Sul	S	0,664	10.408.540	68	6,53	×	0,801	11.377.239	74	6,50
Goiás	CO	0,615	5.210.335	17	3,26	×	0,774	7.020.904	29	4,13
Mato Grosso	CO	0,601	2.604.742	7	2,69	×	0,779	3.484.466	11	3,16
Mato Grosso do Sul	CO	0,613	2.140.624	6	2,80	×	0,777	2.778.986	13	4,68
Distrito Federal	CO	0,725	2.145.839	14	6,52	×	0,859	3.012.718	23	7,63
Brasil		0,692	174.632.960	560	3,21	×	0,765	210.147.125	806	3,84

Abreviaturas – N: Norte; NE: Nordeste; SE: Sudeste; S: Sul; CO: Centro-Oeste; NA: Não disponível.

Os dados foram agrupados por macrorregião brasileira e adicionados a outras variáveis (proporção de centros/pmp, prevalência estimada de pacientes em diálise/pmp/região e mediana do IDH-M/região), que serviram de base para a Tabela 3.

**Tabela 3:** Comparação entre dados demográficos, distribuição de centros de diálise, prevalência de pacientes com drc 5-d em diálise de manutenção e valores de idh-m por macrorregião brasileira entre 2002 e 2019

Macrorregiões	Ano Base 2002	Ano Base 2019	Variação (%)
Norte			
População (n – %)	13.157.728 (7,7%)	18.430.980 (8,8%)	40,08
Centros de diálise (n – %)	13 (2,3%)	47 (6,1%)	261,54
Centros de diálise/pmp	0,99	2,55	NA
Prevalência de DRC 5-D/pmp	109	423	288,07
IDH-M (mediana)	0,525	0,737	NA
Nordeste			
População (n – %)	48.845.112 (28%)	57.071.654 (27,2%)	16,84
Centros de diálise (n – %)	85 (15,2%)	159 (18,6%)	87,06
Centros de diálise/pmp	1,74	2,78	NA
Prevalência de DRC 5-D/pmp	211	594	181,52
IDH-M (mediana)	0,512	0,713	NA
Sudeste			
População (n – %)	74.447.456 (42,6%)	88.371.433 (42%)	18,70
Centros de diálise (n – %)	285 (50,9%)	374 (46,4%)	31,23
Centros de diálise/pmp	3,83	4,23	NA
Prevalência de DRC 5-D/pmp	403	763	89,33
IDH-M (mediana)	0,65	0,801	NA
Sul			
População (n – %)	25.734.253 (14,7%)	29.975.984 (14,2%)	16,48
Centros de diálise (n – %)	133 (23,7%)	157 (19,5%)	18,05
Centros de diálise/pmp	5,17	5,24	NA
Prevalência de DRC 5-D/pmp	362	627	73,20
IDH-M (mediana)	0,664	0,807	NA
Centro-Oeste			
População (n – %)	12.101.540 (7%)	16.297.074 (7,8%)	34,67
Centros de diálise (n – %)	44 (7,9%)	76 (9,4%)	72,73
Centros de diálise/pmp	3,64	4,66	NA
Prevalência de DRC 5-D/pmp	285	743	160,70
IDH-M (mediana)	0,614	0,778	NA
Brasil			
População (n – %)	174.632.960 (100%)	210.147.125 (100%)	20,34
Centros de diálise (n – %)	560 (100%)	806 (100%)	43,93
Centros de diálise/pmp	3,21	3,83	NA
Prevalência de DRC 5-D/pmp	306	665	117,32
IDH global	0,699	0,765	NA

Abreviaturas – IDH-M: Índice de Desenvolvimento Humano Municipal; pmp: por milhão de pessoas; NA: Não Aplicável.

Durante esse período de 17 anos, a variação nacional na prevalência de pacientes em diálise/pmp foi muito superior à variação no número de unidades de diálise (306 para 665 pacientes/pmp – 117,3% vs. 560 para 806 centros – 43,9%, respectivamente) (Tabela 3).

A Região Norte do Brasil apresentou o maior crescimento populacional (13.157.728 vs. 18.430.980 – 40,1%), o maior aumento no número de centros de diálise (13 vs. 47 – 261,54%) e a maior elevação na prevalência de pacientes DRC 5D/pmp (109 vs. 423 pmp – 288,1%). Também apresentou a melhora mais significativa na mediana do IDH-M, de 0,525 para 0,737 (Tabela 3).

No entanto, as mudanças ocorridas nesse período de 17 anos não alteraram geograficamente o percentual de centros de diálise por regiões. O número de centros de diálise/pmp manteve-se concentrado em 2019, especialmente nas regiões Sudeste e Sul (4,23 e 5,24 unidades/pmp), em detrimento das regiões Norte e Nordeste (2,55 e 2,78 unidades/pmp, respectivamente) (Tabela 3).

A distribuição geográfica dos centros de diálise no Brasil em 2019 é apresentada na Tabela 4. Os estados brasileiros estão listados em ordem crescente de valores do IDH-M, com uma divisão por estado das seguintes variáveis: número de centros de diálise, população, número de municípios, municípios com serviços de diálise (total e percentual), centros de diálise nas capitais (total e percentual) e proporção de centros de diálise/pmp (Tabela 4).

**Tabela 4:** Distribuição geogrãfica dos centros de diãlise em todo o território nacional de acordo com os valores crescentes do idh-m (2019)

Estado	IDH-M 2019	Centros de diálise	População (n)	Municípios (n)	Municípios com serviços de diálise (n – %)	Centros de diálise nas capitais (n – %)	Centros de diálise (pmp)
Alagoas	0,687	*14*	*3.336.911*	*102*	*4 (3,9%)*	*10 (71,4%)*	*4,2*
Maranhão	0,694	*13*	*7.075.181*	*217*	*5 (2,3%)*	*7 (53,8%)*	*1,8*
Pará	0,704	*24*	*8.602.865*	*144*	*11 (7,6%)*	*12 (50%)*	*2,8*
Sergipe	0,705	*3*	*2.298.902*	*75*	*1 (1,3%)*	*3 (100%)*	*1,3*
Piauí	0,706	*10*	*3.272.447*	*224*	*5 (2,2%)*	*6 (60%)*	*3,0*
Paraíba	0,713	*11*	*4.018.127*	*223*	*3 (1,3%)*	*6 (54,5%)*	*2,7*
Bahia	0,718	*40*	*14.872.858*	*417*	*24 (5,7%)*	*13 (32,5%)*	*2,7*
Amazonas	0,726	*5*	*4.144.597*	*62*	*1 (1,6%)*	*5 (100%)*	*1,2*
Rondônia	0,73	*7*	*1.777.225*	*52*	*5 (9,6%)*	*3 (42,8%)*	*3,9*
Amapá	0,737	*3*	*845.731*	*16*	*1 (6,2%)*	*3 (100%)*	*3,5*
Acre	0,739	*4*	*881.935*	*22*	*1 (4,5%)*	*4 (100%)*	*4,5*
Pernambuco	0,74	*24*	*9.557.517*	*185*	*11 (5,9%)*	*14 (58,3%)*	*2,5*
Rio Grande do Norte	0,742	*11*	*3.506.853*	*167*	*7 (4,2%)*	*4 (36,4%)*	*3,1*
Ceará	0,744	*24*	*9.132.858*	*184*	*13 (7,1%)*	*11 (45,8%)*	*2,6*
Roraima	0,749	*2*	*605.761*	*15*	*1 (6,7%)*	*2 (100%)*	*3,3*
Tocantins	0,751	*4*	*1.572.866*	*139*	*3 (2,2%)*	*2 (50%)*	*2,5*
Goiás	0,774	29	7.020.904	246	15 (6,1%)	12 (41,4%)	*4,1*
Mato Grosso do Sul	0,777	13	2.778.986	79	7 (8,9%)	6 (46,1%)	*4,7*
Mato Grosso	0,779	*11*	*3.484.466*	*141*	*8 (5,7%)*	*4 (36,4%)*	*3,1*
Espírito Santo	0,793	19	4.018.650	78	9 (11,5%)	4 (21,1%)	*4,7*
Minas Gerais	0,793	93	21.168.791	853	58 (6,8%)	15 (16,15)	*4,4*
Rio Grande do Sul	0,801	74	11.377.239	497	46 (9,2%)	15 (20,3%)	*6,5*
Paraná	0,807	49	11.433.957	399	28 (7%)	13 (26,5%)	*4,3*
Rio de Janeiro	0,809	83	17.264.943	92	30 (32,6%)	41 (49,4%)	*4,8*
Santa Catarina	0,826	34	7.164.788	295	23 (7,8%)	4 (11,8%)	*4,7*
São Paulo	0,845	179	45.919.049	645	84 (13%)	52 (29%)	*3,9*
Distrito Federal^*^	0,859	23	3.012.718	1	1 (100%)	18 (78,2%)	*7,6*
Brasil	0,765	806	210.147.125	5570	405 (7,3%)	289 (50,2%)	*3,8*

Dezesseis (16) dos 27 estados brasileiros apresentaram valores de IDH-M em nível estadual abaixo da média nacional de 0,765, incluindo todos os estados das regiões Norte e Nordeste. Em cinco estados — Sergipe, Amazonas, Amapá, Acre e Roraima — apenas a capital estadual contava com um centro de diálise (Tabela 4).

Com relação aos dados nacionais, apenas 7,3% dos municípios brasileiros (405 de 5.570) possuíam unidades de diálise, em uma proporção de 3,8 centros/pmp. Além disso, mais da metade desses centros (289 unidades; 50,2%) localizava-se nas capitais dos estados (Tabela 4).

A Tabela 5 apresenta a comparação entre os estados com IDH-M inferior (Norte e Nordeste) e IDH-M superior (Centro-Oeste, Sudeste e Sul) ao IDH nacional, com relação às seguintes variáveis: número de centros de diálise, população, número total de municípios, municípios com serviços de diálise, concentração de centros nas capitais estaduais e proporção de centros de diálise/pmp.

**Tabela 5:** Comparação entre a distribuição geográfica e o número de centros de diálise em estados com valores de idh-m acima e abaixo da média nacional

Parâmetros	IDH-M mais baixo (n = 16)	IDH-M mais elevado (n = 11)	Valor de p^*^
Centros de diálise (n – %)	199	607	0,001
População (n – %)	75.502.634	134.644.491	0,036
Municípios (n – %)	2244	3326	0,132
Cidades com serviços de diálise (n – %)	96 (23,7%)	309 (76,3%)	0,003
Número de centros de diálise nas capitais (n – %)	105 (52,7%)	184 (30,3%)	0,001
Número de centros de diálise/pmp	2,8	4,7	<0,001

Nota – *Valor de p pelo teste de *Mann-Whitney*.

As regiões com menor IDH-M (Norte e Nordeste) apresentaram um número significativamente menor de centros de diálise, menos municípios com serviços de diálise, menos centros de diálise/pmp e uma maior concentração de tais centros nas capitais estaduais (Tabela 5).

Observou-se uma correlação linear positiva entre o IDH-M e o número de centros de diálise (coeficiente de correlação de Spearman = 0,52; IC 95%: 0,75–0,18; p = 0,006), conforme demonstrado na Figura 1.

**Figura 1:** Foi encontrada uma correlação moderadamente significativa e positiva entre o escore do Índice de Desenvolvimento Humano (IDH-M) de 2019 e o número de centros de diálise no mesmo ano (coeficiente de correlação de *Spearman* = 0,52; IC 95%: 0,75–0,18; p = 0,006).

## Discussão

As variações demográficas apresentadas na Tabela 1 refletem o envelhecimento acelerado da população brasileira. Esse achado está em consonância com os estudos do *Global Burden of Disease* realizados no Brasil, os quais destacaram o aumento das doenças crônicas não transmissíveis (DCNT) — incluindo a doença renal com necessidade de diálise — como principais contribuintes para a morbimortalidade^21,22,23^. Estima-se que dois terços dos pacientes em TRS no Brasil apresentem como condições subjacentes hipertensão arterial e diabetes mellitus^11,12^.

Esses achados levaram à elaboração do *Plano de Ações Estratégicas para o Enfrentamento das Doenças no Brasil 2011-2022* ^24^ e, mais recentemente, do *Plano de Ações Estratégicas para o Enfrentamento das Doenças Crônicas e Não Transmissíveis (DCNT) no Brasil 2021-2030* ^25^, ambos reforçando a necessidade de criar linhas de cuidado para a vigilância, informação, monitoramento e promoção da atenção integral às DCNT. No entanto, ainda persistem importantes desigualdades tanto no acesso às unidades básicas de saúde quanto no diagnóstico precoce das DCNT, especialmente nas regiões mais vulneráveis do país^26,27^. Estima-se que 10% da população mundial apresente algum grau de perda da função renal; entretanto, uma pesquisa recente revelou que apenas 2% da população brasileira reconhece essa condição, indicando claramente um subdiagnóstico^28^.

Nesse contexto, ainda são necessárias mudanças adicionais no âmbito do SUS para abordar as DCNT, que frequentemente apresentam um curso prolongado e oligossintomático. O sistema deve continuar a se afastar do modelo hospitalocêntrico e curativo — originalmente concebido durante a era do controle das doenças infecciosas — em direção a um novo modelo centrado na promoção da saúde e na prevenção de doenças, baseado em um cuidado contínuo, transdisciplinar e em equipe^29^.

As drásticas mudanças demográficas relacionadas ao envelhecimento não foram acompanhadas por um maior percentual de investimento direto em saúde pública. O crescimento da população idosa nas sociedades contemporâneas gera enormes desafios que precisam ser considerados durante a formulação e o planejamento de políticas públicas estaduais; além disso, a promoção, a prevenção e o cuidado devem ser direcionados às necessidades específicas dessa população, construindo uma rede que possa oferecer serviços e ações de proteção social^30^.

Além dos investimentos limitados em saúde, o período de 17 anos não apresentou melhora na distribuição equitativa de renda e educação (medida pelo IDH global), apesar do crescimento econômico (refletido no aumento do PIB). Entre os países com sistemas universais de saúde pública, o Brasil apresenta um dos menores percentuais de gastos públicos em relação ao PIB, o que tem se mantido ao longo do tempo e fornece argumentos para o mantra utilitarista de que os sistemas universais de saúde representam, na realidade, um “obstáculo” ao crescimento econômico^31^.

O IDH é uma medida sintética de progresso a longo prazo em três dimensões básicas do desenvolvimento humano. Foi criado para ampliar a visão econômica reducionista do desenvolvimento fornecida pelo PIB per capita. Assim, embora ainda seja alvo de críticas, ele inclui educação e saúde, além da renda. Esse índice, portanto, constitui um contraponto ao PIB e é amplamente utilizado como indicador^19^.

As Tabelas 2, 3, 4 e 5 e a Figura 1 foram elaboradas e desenhadas com base nos valores estaduais do IDH-M. Dada a dimensão continental do Brasil e suas significativas disparidades regionais, o Programa das Nações Unidas para o Desenvolvimento no Brasil (PNUD-Brasil), em parceria com o Instituto de Pesquisa Econômica Aplicada (IPEA) e a Fundação João Pinheiro, desenvolveu o IDH-M para avaliar o desenvolvimento humano em nível municipal em todo o país^19^. O cálculo do IDH-M é um ajuste metodológico do IDH global, seguindo suas mesmas três dimensões — longevidade, educação e renda — com base em dados nacionais^19^.

A Tabela 3 mostra que, entre 2002 e 2019, o número de centros de diálise aumentou de 560 para 806 unidades, o que corresponde a um aumento de 43,9%. No mesmo período, prevalência de pacientes DRC 5D/pmp aumentou de 306 para 665, um aumento de 117,3%. Embora ambos os indicadores tenham aumentado, a expansão dos centros de diálise foi proporcionalmente menor do que o aumento na prevalência de pacientes.

Além disso, o crescimento do número de centros de diálise nesse período de 17 anos foi insuficiente para reverter as incontestáveis assimetrias regionais (Tabela 3), com predominância de unidades de diálise e transplante renal nas regiões Sudeste e Sul, em detrimento das regiões Norte, Nordeste e Centro-Oeste. Essa assimetria não é puramente demográfica; embora a região Nordeste seja a segunda mais populosa do país, ela possui menos centros do que a região Sul, evidenciando, assim, a natureza econômica da distribuição dos centros.

O período de 2002 a 2019 foi marcado por um crescimento econômico significativo, mas também por crises financeiras e instabilidade política, especialmente entre 2014 e 2016. No que se refere às políticas de saúde, houve uma expansão progressiva da atenção primária (por meio de programas como a Estratégia Saúde da Família e o Programa Mais Médicos) e avanços na vigilância sanitária e no controle de doenças endêmicas. A expansão dos serviços públicos ocorreu concomitantemente com o fortalecimento do setor privado, limitando a consolidação de um Sistema Único de Saúde verdadeiramente universal e perpetuando o subfinanciamento e as desigualdades^32^.

Uma das explicações para o pequeno crescimento no número de centros de diálise é, sem dúvida, o subfinanciamento crônico do sistema. Entre 2002 e 2017, o valor pago pelo SUS por sessão de hemodiálise aumentou em R$ 91,26 (de R$ 102,94 para R$ 194,20 por sessão), o que corresponde a uma variação média de aproximadamente 5,8% ao ano, abaixo da maioria dos índices de inflação comumente utilizados no Brasil, como o Índice de Preços ao Consumidor Amplo (IPCA) e o Índice Geral de Preços do Mercado (IGP-M)^15^.

Os dados das Tabelas 4 e 5 demonstram uma grave assimetria na distribuição geográfica dos centros de diálise em todo o país, de acordo com os valores crescentes do IDH-M para o ano de 2019. Cinco estados (Roraima, Amapá, Acre, Amazonas e Sergipe) oferecem tratamento de diálise apenas em suas capitais; além disso, em Roraima, existem somente dois centros de tratamento.

A Tabela 5 apresenta a comparação entre os estados com valores de IDH-M superiores e inferiores ao IDH nacional e a evidente desigualdade. Os estados com valores de IDH-M mais baixos (principalmente estados do Norte e Nordeste) apresentam estatisticamente menos centros de diálise, menos municípios com serviços de diálise, menos centros de diálise/pmp e uma maior concentração de centros nas capitais estaduais, corroborando que a disparidade geográfica se manteve ao longo do período de 17 anos do estudo. A Figura 1 mostra uma correlação linear positiva e significativa entre os valores crescentes do IDH-M (mais elevados nas regiões Sul e Sudeste) e uma maior oferta de centros de diálise.

O primeiro Censo Brasileiro de Diálise anual foi instituído pela Sociedade Brasileira de Nefrologia em 1999^13^. No entanto, o primeiro estudo que demonstrou as desigualdades na oferta de tratamento dialítico no país e sua correlação direta com o PIB estadual foi o de Romão-Júnior, que analisou o Censo Brasileiro de Diálise de 2002^10^. Artigos subsequentes analisaram questões específicas relacionadas às disparidades geográficas interestaduais no acesso à diálise^33^. Existem desigualdades na oferta de transplante renal^34^, disparidades espaciais na oferta de equipamentos de saúde (como máquinas de diálise, equipamentos de ressonância magnética e densitômetros ósseos)^35^ e até mesmo diferenças na sobrevida entre pacientes submetidos à diálise pelo SUS e aqueles em diálise por planos de saúde privados^36^. O uso da ferramenta IDH-M não é novidade em estudos brasileiros, os quais demonstram a correlação do índice com a prevalência de doenças infecciosas^37,38,39^ ou mesmo DCNT^40^.

Este é um dos primeiros estudos brasileiros a analisar de forma aprofundada as desigualdades na oferta de diálise, revelando mudanças demográficas, econômicas e sociais relacionadas ao subfinanciamento do SUS durante um período significativo (17 anos). Os valores do IDH-M foram utilizados como ferramenta de análise para demonstrar a manutenção temporal da desigualdade na oferta de diálise no país.

Uma das limitações deste estudo é a utilização dos dados do Censo Brasileiro de Diálise como ferramenta de análise^20^. É importante destacar que os dados são baseados nos centros registrados na Sociedade Brasileira de Nefrologia (SBN), que não constitui um sistema de notificação obrigatória. Portanto, o número real de centros pode divergir, com a possibilidade de subestimação (devido à ausência de registro) ou superestimação (caso os centros tenham encerrado suas atividades, mas não tenham sido removidos do banco de dados).

De fato, como os dados se baseiam em uma pesquisa voluntária preenchida pelos centros de diálise, a prevalência calculada é aproximadamente igual ao número de pacientes em diálise, não individualizada, e frequentemente sujeita a subnotificação, por não ser obrigatória. Essa situação incentivou alguns pesquisadores a utilizar outras fontes de dados, como a emissão da APAC/TRS (Autorização de Procedimentos de Alta Complexidade/Terapia Renal Substitutiva)^41^.

Um artigo recente, utilizando dados da base de 2023 do Sistema Único de Saúde, complementa os dados de nosso estudo ao apontar que - em um cenário pós-COVID - pacientes das regiões Norte, Nordeste e Centro-Oeste percorrem distâncias maiores para realizar o tratamento de hemodiálise em comparação aos pacientes das regiões Sul e Sudeste^42^.

## Conclusão

Observaram-se mudanças significativas nos aspectos sociodemográficos brasileiros no período analisado (2002 a 2019), especialmente no que se refere ao crescimento da população idosa. No entanto, tais mudanças não se traduziram em um aumento no percentual do PIB destinado à saúde ou, ainda menos, na oferta equitativa de diálise em âmbito nacional, mesmo diante do claro aumento na prevalência de DRC 5-D. Além disso, as desigualdades regionais são evidentes, com centros de diálise concentrados em regiões de maior renda e em estados com valores mais elevados de IDH-M.

## Disponibilidade de Dados

O conjunto completo de dados que sustenta os achados deste estudo está disponível mediante solicitação ao autor correspondente.
